# Faculty curriculum development to open the gates to STEM: incorporating research experiences to the curriculum to increase student success in STEM

**DOI:** 10.3389/fsoc.2026.1904900

**Published:** 2026-10-01

**Authors:** Heather Haeger, Joseph Owusu Amoah, Enock Atta-Sah Nimfour

**Affiliations:** 1 Department of Educational Policy Studies and Practice, The STEM Learning Center, University of Arizona, Tucson, AZ, United States; 2 Department of Educational Policy Studies and Practice, University of Arizona, Tucson, AZ, United States

**Keywords:** course-based undergraduate research experiences, curriculum development, gatekeeping STEM courses, retention, student success

## Abstract

**Introduction:**

As access to college has increased, the access point has shifted from getting into college to persisting in elite majors, such as STEM majors which provide access to greater economic mobility. Passing through introductory gatekeeping courses in STEM, courses with high rates of dropping, failing, withdrawing, or taking an incomplete (DFWI rates), is a crucial access point for undergraduate students. Being able to pass these courses is a high-stakes filter that has a significant influence on persistence, retention, and degree completion in college. This study examines an intervention designed to improve student success and retention in STEM by supporting curriculum re-design of gatekeeper courses through Course-Based Undergraduate Research Experiences (CUREs). CUREs offer students opportunities to engage in authentic research, which provides students with practical experience to develop collaboration, communication, teamwork, problem-solving, and other competencies that help them succeed in gatekeeper courses in the early stages of the STEM pathways.

**Methods:**

We employ a *post hoc*, quasi-experimental design to test the impact of this curriculum re-design on college persistence, persistence in STEM, and cumulative GPA. The study was conducted at a large public research university designated as a Hispanic-Serving Institution. The treatment variable indicated participation in a CURE designed through the CURE Training Institute, a 1-week intensive training for STEM faculty teaching introductory STEM courses, followed by a year-long Community of Practice. Outcome variables included first- and second-year college retention, first- and second-year STEM retention, and cumulative GPA at the end of the second year. To address selection bias, Propensity Score Matching was used to create comparable groups of students who did and did not participate in CUREs. Regression analyses were then conducted on both full and matched samples to estimate the relationship between CURE participation and academic outcomes.

**Results:**

Using propensity score matching, the results show a consistent pattern in which participation in course-based undergraduate research experiences is associated with significantly higher retention in college and STEM retention.

**Discussion:**

Integrating faculty curriculum development through course-based undergraduate research experiences supports students in succeeding in gateway courses without lowering academic rigor and the knowledge and mastery students need to possess.

## 1 Introduction

Students in science, technology, engineering, and mathematics (STEM) take introductory courses, such as intro to biology or general chemistry, in their first terms of their programs (Hatfield et al., 2022). Koch (2017) defines gateway courses as foundational, high-enrollment, and high-risk courses that serve as critical entry points into academic programs. These courses are characterized by high rates of dropping, failing, withdrawal, or taking an incomplete (DFWI) and enroll large numbers of students, making them critical to student persistence and degree completion in higher education. These introductory courses with high DFWI grades are also referred to as “weed-out” or “gatekeeping” courses (Weston et al., 2019). These courses serve as a means of imparting base knowledge in STEM disciplines but also serve to separate students who demonstrate persistence and academic capacity to succeed in STEM from those perceived as less likely to persist. As a result, introductory courses serve as a sorting function that reinforces their designation as “gatekeeping” within STEM education (Thompson, 2021). Though the espoused function of this gatekeeping is to sort students based on merit and ability, in actuality, students who are minoritized or stigmatized based on their race, gender, socioeconomic status, and disability status can also be sorted out of STEM majors (Seymour and Hunter, 2019). This has been demonstrated even when controlling for academic preparation and academic aptitude (Hatfield et al., 2022; Weston et al., 2019). Many undergraduate STEM programs experience low academic engagement in introductory STEM courses, which can contribute to reduced persistence and increased switching out of science majors. Disengagement in introductory STEM courses is often identified as a primary reason students leave STEM majors, particularly when instructional support is not flexible, authentic, or engaging, and when instructors do not consistently support all learners, including racially minoritized and other underserved student populations (Seymour and Hunter, 2019). In contrast, students report higher engagement when they feel comfortable asking questions in class, seeking tutoring, attending supplemental instruction sessions, and collaborating with peers (Gasiewski et al., 2012).

One strategy to increase engagement and address the high DFWI rates in introductory STEM courses is curriculum development to incorporate Course-Based Undergraduate Research Experiences (CUREs) into STEM gateway courses. The core tenets of CUREs represent a pedagogical shift that embeds authentic research into credit-bearing coursework and expands access to undergraduate research opportunities for a wider range of students (Corwin et al., 2018). Unlike traditional laboratory courses with predetermined outcomes, CUREs engage students in open-ended inquiry, data analysis, and collaboration that mirror professional research practices. CUREs offer institutions the opportunity to integrate undergraduate research at scale while maintaining academic rigor and relevance. Introductory courses in STEM programs are often framed as mechanisms for distinguishing students who are perceived as able to meet the academic rigor of a STEM major. However, students' experiences in these courses and the extent to which they demonstrate mastery of disciplinary knowledge do not affect all students equally across demographic groups. Grades in gatekeeping courses can differ systematically by race, gender, and other student characteristics (Eddy et al., 2014), and students' responses to these grades can also vary based on multiple factors, including prior preparation and the availability of academic and social support. Prior studies have shown that students participating in CUREs report gains in scientific self-efficacy, research skills, and interest in STEM fields (Rodenbusch et al., 2016). Our study seeks to explore whether redesigning introductory STEM courses with higher DFWI rates to be CUREs can increase persistence in STEM. Faculty professional development served as the institutional approach to designing and implementing CUREs across gateway STEM courses. Although this study does not explicitly evaluate faculty's professional development, it establishes the implementation context within which student outcomes associated with CURE participation are examined.

The purpose of this study is to examine how CUREs promote students' success, retention, and persistence in STEM pathways by integrating faculty development interventions that embed CUREs in early gateway introductory courses and support students through degree completion. Kuh (2008) includes Undergraduate Research Experiences (UREs) among HIPs, noting their ability to promote reflective, engaged learning that extends beyond academic performance into civic and professional life. CUREs represent a pedagogical shift by embedding genuine research experiences into standard coursework (Corwin et al., 2018). While traditional research opportunities are often limited to select students, CUREs democratize access by allowing all participants in a course to take part in meaningful, hands-on inquiry (Rowland et al., 2012). These experiences actively involve students in the processes of scientific reasoning, data interpretation, and problem-solving, strengthening their academic involvement while simultaneously fostering skills essential for career development (Auchincloss et al., 2014). Majka et al. (2023) highlight CUREs as a resource-efficient and scalable strategy for expanding undergraduate access to research experiences. Their contribution to workforce readiness stems from their emphasis on authentic research practices that mirror those used in professional environments. In contrast to conventional laboratory courses where outcomes are predetermined, CUREs immerse students in inquiry-driven projects where results are uncertain. This environment cultivates discovery and analytical thinking, while also building transferable competencies like critical thinking, persistence, and data literacy (Duschl et al., 2007). These research experiences also foster communication, collaboration, and leadership as students engage closely with peers, teaching assistants, and faculty members (Singer et al., 2006). The structure of CUREs encourages feedback, iteration, and adaptation, allowing students to refine their research approaches and develop resilience in the face of challenges. Importantly, because many CURE projects produce findings that extend into real-world scientific or societal contexts, students gain a deeper understanding of the relevance and potential impact of their contributions, which supports their developing professional identity (Quinn et al., 2011).

UREs are recognized as high-impact practices that enhance research skills, STEM persistence, and graduation outcomes (Kuh, 2008; Dong et al., 2024; Hunter et al., 2007; Russell et al., 2007). The features that define CUREs position them as high-impact practices that not only enhance learning in STEM programs, but these experiences help students build cognitive, technical, and interpersonal competencies aligned with national career readiness frameworks like those of National Association of Colleges and Employers (2024) which serves an integral STEM pathway (Kuh, 2008). The open-ended and collaborative nature of CUREs help students to develop scientific reasoning, data analysis skills, and critical thinking (Corwin et al., 2015). Numerous studies have shown that students who participate in CUREs report gains in self-confidence, scientific identity, and sustained interest in STEM fields (Lopatto, 2007). Despite their many strengths, several gaps remain in the literature on CUREs. As Majka et al. (2023) observe, common methodological limitations include volunteer bias, small sample sizes, and the absence of control groups for comparison (Brownell et al., 2013; Flannelly et al., 2018). This study addresses such concerns by utilizing a larger and more diverse sample of students, along with propensity score matching to create a control group of like-peers that simulate random assignment to a treatment and control group.

This study utilizes data from students who were in a CURE developed through the CURE Training Institute. The Institute focused on the following key components of a CURE (National Association of Colleges and Employers, 2024):

- “CUREs offer opportunities for students to make discoveries that are of interest to stakeholders outside the classroom (e.g., the broader scientific community).
- Students' work is iterative, meaning that students must trouble-shoot, problem-solve, and repeat aspects of their work for the research to progress.
- CUREs offer opportunities for students to communicate their research results to those stakeholders.
- Just like in a faculty member's research group, the research in a CURE progresses as students work. This means that new research questions and directions are generated each term, and the CURE is unlikely to look the same from year to year.
- Students may engage in a range of science practices such as collecting and analyzing data, building and defending arguments, and collaborating with one another and more experienced scientists. The work that students do in a CURE must build off and have the potential to contribute to a larger body of knowledge in the discipline.”

Faculty in the Institute report that CUREs are an effective pedagogical approach for fostering student success in STEM. Participants in the CURE Training Institute reported high levels of satisfaction with the program (M = 9.5/10; Kahlid et al., 2025). All participants reported feeling more confident and better prepared to teach a CURE course after learning about backward design, inclusive course design, potential implementation challenges, and strategies shared by experienced CURE instructors. Faculty also highlighted the effectiveness of the program's interactive format, including hands-on activities and collaborative discussions. While these findings suggest that faculty perceive CUREs as effective for promoting student learning, engagement, and retention in STEM, this study tests the actual impact of CUREs on student success. Specifically, the study examines whether participation in CUREs is associated with improved STEM persistence and academic performance.

This study investigates how CUREs function, offering insights into how research experiences can strengthen student success and enhance retention. In addition, most assessments of CUREs depend on student self-reports rather than using more objective measures (Chase et al., 2016; Haeger et al., 2020). Consequently, there is limited empirical evidence linking CUREs with academic success and persistence. In STEM programs, poor performance in introductory courses can reduce students' interest in STEM, leading them to drop out or switch to non-STEM majors (Theobald et al., 2020; Thompson, 2021). In a study by Thompson (2021), it was found that grades in first-year courses do not affect students evenly, which supports the idea that gatekeeping courses push some students out of STEM. The study also examined STEM persistence among first-generation and continuing-generation students. Even among students with high GPAs, first-generation students were less likely than their continuing-generation peers to persist in STEM. In this context, the tenets of CUREs can promote persistence and equity when they are implemented intentionally, to support underserved students and help them successfully pass gatekeeping introductory courses. This study seeks to examine how curriculum development to implement CUREs foster more equitable success in STEM through the following question:

- Does participation in a CURE improve student success as measured by first- and second-year retention in college, first- and second-year retention in STEM fields, and cumulative GPA when controlling for student background characteristics and early academic performance and when compared to like-peers?

We use institutional data to conduct a *post-hoc*, quasi-experimental study answering this question.

## 2 Literature review

Student experiences and academic performance in their first year are extremely influential in their overall academic trajectory and for their persistence in STEM (Seymour and Hunter, 2019). Students who earn higher GPAs during their first year in STEM programs are more likely to persist in STEM majors (Seymour and Hewitt, 1997). In contrast, students with lower first-year GPAs are often discouraged from continuing in STEM and may switch to other fields. Flanders (2017) examined the relationship between gateway course completion and first-year student retention. Students who successfully completed a gateway course within their declared major during their first semester were more likely to persist in the second semester than those who did not complete the course or delayed taking it. It was further argued that completing a gateway course early may strengthen students' commitment to their academic major and support retention during the first year of college. The study affirms that gateway courses are an important part of student persistence through STEM pathways in higher education. Moreover, (Koch and Drake 2018) found that gateway STEM courses are significant to student success because they influence persistence and degree completion.

In recent years, authentic research approaches like CUREs offer undergraduate students' opportunities to fully engage in research and have proven to be an effective pedagogical approach in undergraduate education (Corwin et al., 2018). Unlike traditional laboratory research that follows predetermined outcomes, CUREs engage in authentic and novel inquiry that reflects the complexities of real-world scientific investigation (Brownell and Kloser, 2015). CUREs have proven to provide several advantages over traditional laboratory courses and research internships for undergraduate students (Corwin et al., 2015). As higher education institutions make significant efforts to improve STEM education, CUREs have emerged as an effective and promising approach for equipping students with career-relevant skills fostering student success in their course of study and in the workforce after graduation (Rodenbusch et al., 2016). The value of CUREs has been demonstrated across disciplines. In a study, Broussard et al. (2025) found that participation in CUREs resulted in significant gains in students' scientific practice skills and more positive research self-perceptions. These findings indicate that CUREs strengthen core research competencies and provide meaningful learning experiences for students in both the natural and social sciences.

Cooper and Brownell (2018) argue that CUREs combine teaching and research by engaging entire classes in projects that generate new, externally relevant data, achieving success by targeting both pedagogical outcomes (e.g., students' ability to collect and analyze data and to communicate findings) and tangible research outputs (e.g., posters or publications). The structure of CUREs supports the development of technical skills and content knowledge, but their value extends far beyond the laboratory. Students in CUREs report greater confidence in their scientific abilities and stronger identification with the scientific community (Bangera and Brownell, 2014). As a result, these experiences are crucial, as self-efficacy has been linked to persistence and success among students in STEM programs.

Studies have further highlighted that skill development is an essential impact of CUREs. Ballen et al. (2017) found that participation in a biology CURE led to measurable increases in students' quantitative reasoning and data interpretation skills. Meanwhile, (Cooper and Brownell 2018) documented improvements in collaborative problem-solving and scientific writing among students in a chemistry-focused CURE. These studies underscore that CUREs are not just a content-delivery approach, but dynamic environments where students learn to think, write, and work together, leading to their success and retention in STEM programs. Dolan and Weaver (2021) emphasized that well-designed CUREs promote structured collaboration, which not only mirrors real scientific research but also cultivates interpersonal skills essential in professional settings. Likewise, studies in psychology and neuroscience CUREs have shown that students develop a stronger sense of peer accountability and group efficacy when working on open-ended projects with real consequences (Shortlidge and Brownell, 2016).

Much literature on CUREs shows that biology and chemistry are prominent in the CURE literature; however, CUREs in other disciplines and interdisciplinary applications are expanding. Psychology-based CUREs, for instance, often focus on experimental design, statistical analysis, and ethics, which are skills that translate directly to clinical and social science careers (Haeger et al., 2020; Wayment and Dickson, 2008). In neuroscience, students working in lab-based CUREs have reported improvements in data visualization and computational modeling, aligning with the growing emphasis on data fluency in health and tech industries. This interdisciplinary application of CUREs shows the flexibility of the CURE model, and the underlying structure of engaging in authentic, collaborative research builds a shared skill set that supports diverse career paths. Newer interdisciplinary programs, such as those described by Majka et al. (2023), demonstrate the feasibility of integrating lab-based CUREs across environmental science, engineering, and health-related fields, allowing students to gain cross-sector technical competencies. In conclusion, CUREs offer students hands-on experience with research, foster a range of transferable skills like teamwork, collaboration, problem-solving, and communication skills, and provide inclusive access to high-impact learning. Our study will further test the impact of implementing CUREs into early career STEM courses to improve retention and success in STEM.

According to Laporte and Casper (2026), institutional context and selection bias are important considerations when interpreting CURE outcomes. Using institutional data from more than 2,500 students across multiple semesters, the authors found that the positive effects of an introductory biology CURE were evident only when students voluntarily enrolled. When participation was mandatory, however, the CURE neither improved institutional retention nor increased subsequent biology enrollment compared with traditional laboratory courses. Instead, concurrent GPA emerged as the strongest predictor of student persistence, suggesting that student characteristics and institutional context substantially influence CURE outcomes. Similarly, Linn et al. (2015) cautioned that findings from undergraduate research experiences are often confounded by volunteer bias because students who choose to participate typically have stronger academic preparation, higher motivation, and greater interest in science than their peers. We build on these studies by addressing the need for more rigorous study designs that incorporate objective institutional outcomes, creating an appropriate comparison group, and controlling for early academic performance.

Mashintonio and Heineman (2025) further challenged the assumption that CUREs consistently improve student outcomes by comparing an introductory biology CURE with a traditional laboratory course. Contrary to much of the existing literature reporting positive effects of CUREs, they found that the CUREs had minimal effects on students' academic performance, self-confidence, and laboratory preferences and did not improve 1-year retention. These findings highlight the importance of evaluating CUREs using objective outcomes and rigorous research designs and suggest that the effectiveness of CUREs may depend on institutional context, implementation, and student characteristics rather than the research experience itself. Our study addresses these methodological gaps by employing propensity score matching and objective institutional data to reduce bias arising from observed differences between CURE participants and non-participants. This approach provides a more rigorous evaluation of the association between CURE participation and student success outcomes, including cumulative GPA, first- and second-year retention, and STEM persistence.

Faculty development is critical to the successful design and implementation of CUREs. Zilvinskis et al. (2023) argue that professional development is essential for building faculty and staff capacity to implement HIPs equitably and effectively. Therefore, professional development should be proactive, practical, and inclusive to help educators better understand and address barriers to student success while supporting broader institutional efforts across the curriculum. Hosein and Rao (2017) found that CUREs integrating student-centered learning with purposeful faculty guidance produced better educational outcomes, emphasizing the importance of faculty development in CURE design. Early research on faculty experiences with CUREs advanced understanding of the conditions necessary for successful implementation. (Shortlidge and Brownell, 2016) showed that, although faculty valued CUREs for integrating teaching with research and expanding students' access to authentic research experiences, implementation was constrained by demands on time, resources, course management, and the uncertainty inherent in authentic research. Drawing on the perceptions of faculty across diverse institutional contexts, the study further identified research expertise, instructional flexibility, sustained time investment, and institutional support as critical conditions for effective CURE implementation. These findings reveal that faculty preparation and institutional support are central to the successful design and delivery of CUREs. Gough et al. (2025) extended the work on faculty development for CUREs by demonstrating how sustained professional development supports both CURE implementation and broader instructional change. Drawing on the experiences of 11 biology faculty members, the authors found that peer learning, expert facilitation, and participation in a community of practice shaped faculty members' CURE design, inclusive teaching practices, and the integration of research into the classroom. The study further showed that sustained collaboration and structured reflection enabled faculty to refine their instructional practices beyond the initial implementation of a CURE, suggesting that ongoing professional development is essential for continuous pedagogical improvement. Norman et al. (2024) examined how institutions can use professional development to support CURE implementation and long-term sustainability. The authors described a cohort-based professional development model that integrated CURE pedagogy, inclusive teaching, peer mentoring, and communities of practice to address barriers to CURE implementation. They argued that sustained institutional support, including workload accommodations, collaborative learning, leadership engagement, and communities of practice, is essential for overcoming implementation barriers and sustaining CUREs beyond external funding. The study highlights the role of institutional capacity, in addition to faculty preparation, in supporting the successful implementation and long-term sustainability of CUREs. Soto et al. (2025), in a perspective article, examined the professional value of CURE teaching for faculty. Drawing on the implementation of three biochemistry CUREs, the authors argued that, despite the substantial time, preparation, and instructional effort required, CUREs can enhance faculty scholarship by generating preliminary data, supporting publications and grant proposals, and recruiting students into research laboratories. They also identified intellectual stimulation and renewed engagement with teaching and research as important professional benefits. The authors further argued that sustained institutional support, including professional development, mentoring, and administrative resources, is essential for the long-term implementation of CUREs. The article suggests that the continued expansion of CUREs depends not only on evidence of student benefits but also on institutional recognition and support for the scholarly and instructional contributions of faculty. Parks and Meitzen (2026) emphasize that faculty professional development is fundamental to expanding and sustaining CURE implementation. They found that collaborative workshops, faculty mentoring, and shared course design increased faculty capacity to develop CUREs, strengthened cross-disciplinary collaboration, and enhanced institutional capacity to scale CUREs across STEM programs.

## 3 Theoretical framework

This study is grounded in Kolb's (1984) Experiential Learning Theory (ELT) and Tinto's Model of Student Retention (2012, 2017). Kolb's (1984) ELT posits that learning occurs through the transformation of experience. The theory consists of four interconnected stages: concrete experience, reflective observation, abstract conceptualization, and active experimentation. Effective learning occurs when students engage in each stage of this continuous cycle. Because CUREs engage students in authentic research, they provide opportunities to investigate real-world problems, analyze data, reflect on findings, and apply new knowledge. These experiences promote critical thinking, knowledge construction, and deeper learning, making ELT an appropriate framework for understanding how CUREs contribute to student success.

Tinto's Model of Student Retention (Tinto, 2012, 2017) explains how institutional practices and meaningful educational experiences promote student persistence. Tinto argues that students are more likely to remain enrolled when institutions provide supportive learning environments that foster academic engagement, meaningful interactions with faculty and peers, and a strong commitment to students' educational success. Within this framework, CUREs represent an institutional commitment to strengthening academic integration by engaging students in authentic research embedded within gateway STEM courses, thereby promoting student success.

These frameworks support this study by providing a solid foundation for understanding how CUREs may influence student success. ELT explains how students develop knowledge and skills through authentic research experiences, while Tinto's model explains how institutional commitment to student success through intentional efforts, such as curriculum redesign, can strengthen academic engagement and persistence. As part of the curriculum redesign of gateway STEM courses, CUREs provide students with sustained opportunities to investigate scientific questions, collaborate with peers and faculty, and communicate original findings (Auchincloss et al., 2014; Corwin et al., 2018; Kleinschmit et al., 2023). Embedding research experiences within required coursework also expands access to learning opportunities that have traditionally been limited to a small group of students, creating more equitable pathways to academic success in STEM (Corwin et al., 2018; Museus and LePeau, 2023).

CUREs are implemented in gateway STEM courses as part of the institution's commitment to student success. Through authentic research, they provide students with opportunities to collaborate, generate new knowledge, and develop ownership of their learning (Tinto, 2012, 2017; Laporte and Casper, 2026). Accordingly, this study draws on ELT to explain how students learn through authentic research experiences and Tinto's Model of Student Retention to explain how institutionally embedded CUREs can promote academic engagement, retention, and STEM persistence.

## 4 Methodology

This study was conducted at the University of Arizona in Tucson, Arizona, a large public research institution. As of Fall 2024, the university reported a total enrollment of 56,365 students, including both graduate and undergraduate populations (Arizona Board of Regents, 2025). The institution is a designated Hispanic-Serving Institution (HSI) with a strong commitment to equity and access for diverse student populations across the United States and beyond.

This study gathered data from 11,124 students at the University of Arizona. The data comprises the entire entering class of 2022 and included 232 students who took a CURE developed through the CURE Training Institute in their first year (Figure 1). Though a higher proportion of STEM students have now taken CUREs, students from later cohorts or who took a CURE in their second or third year were excluded so that we could study retention after year one and year two.

**Figure 1:**
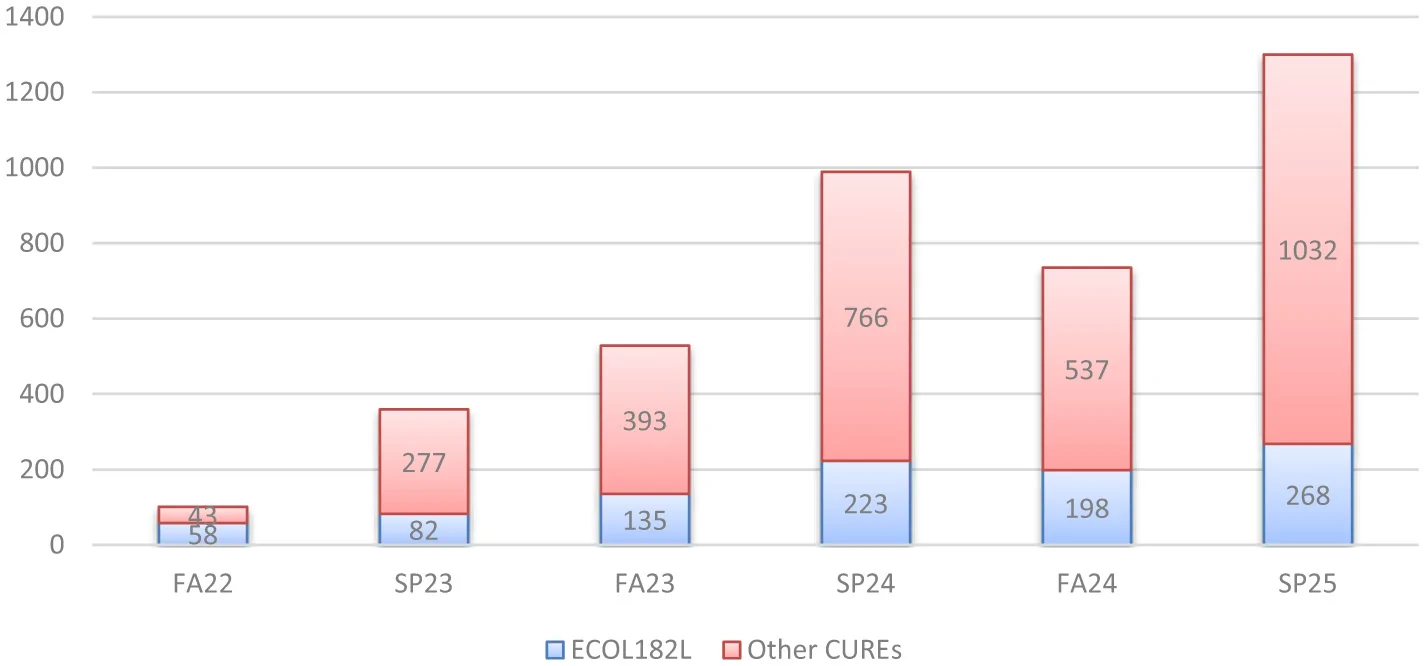
CURE Enrollment (Fall 2022-Spring 2025).

The CURE Training Institute at the University of Arizona offers support for faculty in designing and implementing CUREs through structured training, workshops, and a community of practice. Faculty who taught large, introductory STEM courses were invited to participate in the CURE Training Institute; the largest course being the Introductory Biology I Laboratory (ECOL 181L). Other smaller, introductory STEM courses include Introduction to Biosystems Analytics & Technology (BAT 102), Introduction to Biomedical Research (BIOC 296B), Experiences in Geochronology (GEOS 197E), Tree-ring time machines (GEOS275), and Introductory Physics (PHYS 181) are represented collectively, in red in Figure 1. As part of the curriculum redesign of gateway STEM courses, participating faculty completed a structured professional development program. While faculty participation and professional development outcomes were not evaluated, faculty professional development served as the implementation context for the CUREs examined in this study.

Only sections of each course that were taught by faculty who completed the CURE Training Institute and who confirmed that they were teaching the course as a CURE were included in the sample (Figure 1). All of the courses in the sample were pre-existing, introductory STEM courses redesigned to include authentic research while still covering the approved core course content. The university course listing did not distinguish which courses or sections were taught as a CURE and which were not, so students did not purposefully enroll in a CURE, but enrolled in a required course that happened to also be a CURE. Figure 1 represents all students who completed a CURE designed through the CURE Training Institute between Fall 2022 and Spring 2025. Though more than 4,000 students have taken CUREs designed through the Institute, this study is focused on the 232 students from the enter class of 2022 who took a CURE in Fall 2022 or Spring 2023 (their first year in college). Additional students enrolled in CUREs that year, and in subsequent years, but in order to study persistence in college and in STEM, only first-year students from the 2022 cohort were included in the analysis as the CURE treatment group (232 first-year students out of the 450+ students who took a CURE in academic year 2022).

The source of data for the study was the Office of Institutional Research at the University of Arizona. Student records were de-identified to protect students' privacy. The study was approved by the University of Arizona Institutional Review Board (IRB) (STUDY00001186).

### 4.1 Descriptive statistics

The study used institutional records that included student background characteristics, enrollment information, major, and academic performance. Full-time refers to students who were enrolled in 12 units or more at the time of the study. First-generation students represent students whose parents or guardians do not hold any post-secondary degrees. Pell Grant eligibility is represented by Pell, indicating students who qualify for Pell Grants and thus have financial need. CURE refers to students who took a course developed through the CURE Training Institute. STEM represents students enrolled in a Science, Technology, Engineering, or Mathematics major. 1st semester GPA refers to the GPA attained by students at the end of their first semester. 1st-year retention indicates whether a student continued enrollment into their second year. 2nd-year retention represents whether a student continued into their third year. 1st-year STEM retention indicates whether a STEM major student remained at the university and in a STEM field into their second year. Second-year STEM retention shows whether a STEM major student remained in a STEM field and at the university into their third year. Scond-year GPA refers to the cumulative GPA at the end of the second year.

Table 1 above presents descriptive statistics for the key variables in the study based on a sample of 11,124 students from the University of Arizona. Approximately 70% of students were enrolled full-time, and 33% were first-generation college students. About 27% of the samples were Pell Grant recipients, indicating financial need. 40% of students in the sample were enrolled in STEM majors. The sample has a higher proportion of female students (59%) compared to male students (41%). The sample was limited to a binary gender category and does not account for non-binary students or other gender identities. Table 1 also shows that half the student population was White (51%), and over a quarter were Hispanic or Latine (29%). Smaller proportions of the student population were Black or African American (5%), Asian (5%), those who identify with two or more races (5%) Native American or Alaska Native (1%), International students (3%), and Native Hawaiian or Other Pacific Islander (0.22%). Moreover, 2% of students did not report their ethnicity. Students had an average GPA their first semester of college of 2.72.

**Table 1:** Descriptive statistics of full and matched samples.

Variable	Full sample^a^ (*N* = 11,124)	CURE^b^ (*n* = 232)	Full sample No-CURE^c^ (*n* = 10,892)	Matched sample No-CURE^d^ (*n* = 3906)
STEM	40%	80%	39%^**^	48%^**^
Fulltime	70%	86%	70%^**^	77%^**^
First-generation	33%	27%	33%^**^	25%
Pell	27%	24%	27%	22%
Female	59%	65%	59%	67%
Male	41%	35%	41%	33%
Native American or Alaska Native	1%	0.05%	1%	1%
Asian	5%	7%	5%	5%
Black or African American	5%	1%	5% ^*^	3%
Hispanic or Latine	29%	31%	28%	26%
International Student	3%	2%	3%	2%
Native Hawaiian or Other Pacific Islander	0.2%	0%	0.1%^**^	0%
Two or more races	5%	5%	5%	4%
White	51%	52%	50%	57%
Not reported	2%	1%	2%	2%
1st semester GPA	2.72	3.36	2.70^**^	3.32

a The full sample, including all students in the entering cohort 2022.

b Students from the entering cohort 2022 who took a CURE course in their first or second semester (fall 2022 or spring 2023). The population of CURE students is the same in the full and matched datasets.

c Comparison group of students who did not take a CURE from the full sample. ^*^p < 0.01, ^**^p < 0.001 in *T*-tests comparing the proportion (or mean for GPA) of CURE students compared to students who did not take a CURE in the full sample.

d Matched comparison group of like-peers who did not take a CURE from the matched sample. ^*^p < 0.01, ^**^p < 0.001 in *T*-tests comparing the proportion (or mean for GPA) of CURE students compared to like-peers who did not take a CURE in the matched sample.

*T*-tests were performed to test the differences between students in CUREs and the overall student population who hadn't taken a CURE developed through the CURE Training Institute (Table 1). CURE students were significantly more likely to be in STEM, be enrolled fulltime, and were significantly less likely to be a first-generation college student or be Black or a Pacific Islander. CURE students also had an average first semester GPA of 3.36 which was significantly higher than non-CURE students with a GPA of 2.70.

### 4.2 Outcome variables

The outcome variables of interest in this study are multiple measures of academic success: first-year and second-year college retention, first-year and second-year STEM retention, and cumulative GPA at the end of the second year. These metrics are essential for determining the efficacy of CUREs in maintaining students' interest and dedication to STEM degree programs. STEM retention is a key component as it has direct bearing on the proportion of students who pursue STEM careers.

### 4.3 Treatment variables

The treatment variable indicates students who participated in a CURE. Students in CURE courses conduct research as part of their course work, with the goal of improving their grasp of STEM areas via more experiential, inquiry-based learning. Only students in CUREs developed through the CURE Training Institute and in introductory STEM courses were used in the analysis. Additionally, only students who participated in a CURE in their first year of college were included in the treatment group because students who took a CURE in their 2^nd^ year are also already retained through their 2^nd^ year.

### 4.4 Estimation approach

The purpose of this study is to investigate the impact of CURE courses on academic success when compared to like-peers through Propensity Score Matching (PSM; Byrd et al., 2022). The goal is to establish a balanced sample of students who participated in CURE programs and non-participants who are similar in terms of major, enrollment status, background characteristics, and early academic performance. Propensity score is estimated using a logistic regression model, where the probability of receiving the treatment (T) is modeled as a function of a set of covariates (X; Guo and Fraser, 2014). The equation for the propensity score matching (PSM) can be written as:


$$\begin{array}{l} \text{PS}=\text{P}\left(\text{T}=1|\text{X}\right)=\text{Λ}\left(\beta X\right) \end{array}$$

Where: PS = propensity score (probability of receiving the treatment), T = treatment indicator (1 = received treatment, 0 = did not receive treatment), X = vector of covariates (predictor variables), β = vector of coefficients (parameters) estimated from the logistic regression model, Λ = logistic function, defined as:


$$\begin{array}{l} \text{Λ}\left(x\right)=1/\left(1+e\wedge \left(-x\right)\right) \end{array}$$

The equation can be expanded to include the specific covariates and coefficients, for example: PS = P(T=1|X) = Λ(β0 + β1X1 + β2X2 + … + βkXk); where X1, X2, …, Xk are the covariates, and β0, β1, β2, …, βk are the coefficients estimated from the data. The logistic function maps the linear predictor (βX) to a probability between 0 and 1.

We used a logistic regression model to estimate propensity scores (Guo and Fraser, 2014). This model regresses the treatment variables (CURE) on the covariate. In a STEM program, students might select courses based on various factors. Propensity Score Matching (PSM) helps create comparable groups by matching students in the treatment groups (those who participated in CURE) with students in the control group (those who didn't) based on their propensity score or propensity to participate. We utilized the nearest neighbor matching method to guarantee groups are comparable. This reduces selection bias and allows for a fairer evaluation of the program's impact on retention. To account for selection into treatment, we estimate the propensity score, and match individuals based on their value. The propensity score is the likelihood of treatment based on student demographic characteristics (race/ethnicity, gender, parental education, Pell eligibility), enrollment information (full-time enrollment, major at enrollment), and early academic performance (first semester GPA). Scholars employ PSM and make modeling judgments when using this technique, including various typical matching techniques (Olitsky, 2014). There is a compromise between estimation efficiency and bias for every method. This method makes extensive use of measures to minimize bias. We employed the nearest neighbor with the greatest allowed caliper matching estimator. This method matches students in the treatment group with a control group whose propensity score is within.00001 difference of the treated student's score. Additionally, we utilized the nearest neighbor algorithm with replacement to decrease the bias of the estimates. This allows one person in the control group to match up with many people in the treatment group.

The matched sample substantially reduces the differences in the population of CURE students and the comparison group of like-peers, though there are still some significant differences (Table 1). In testing preliminary models, first semester GPA was the highest predictor of future academic performance, so we found it most important to reduce the difference in early academic performance. In the full sample, CURE students had a 0.66 point higher first semester GPA than students who didn't take a CURE course which was also statistically significant (CURE GPA 3.36 and no-CURE GPA 2.71. In the matched sample, CURE students had a 0.04 difference in first semester GPA and this difference was not significant (CURE GPA 3.36 and non-CURE 3.32). Even in the matched sample, CURE students were still significantly more likely to be in STEM and to be enrolled fulltime.

### 4.5 Data analysis

We employed the nearest neighbor matching method within a Propensity Score Matching (PSM) framework to ensure comparable groups and to minimize selection bias, facilitating a more accurate assessment of the program's impact on retention and academic performance. Propensity estimates for the likelihood of participating in CURE were created and used to create a matched dataset of peers that were equally likely (matched to at least.00001 propensity estimate). Before matching, CURE students had significantly higher first-semester GPA and were more likely to be full-time, Pell eligible, first-generation college students, and STEM majors. In the matched sample, these demographic differences were all reduced, and there was no longer a significant difference in first-semester GPA. Once the matched dataset was created, we conducted a series of regressions to test the relationship between participating in CURE on student success. The variables of interest in this analysis are university retention and STEM retention, and CURE participation. In this analysis, STEM retention variables and GPA serve as our dependent variables, while participation in the CURE course is our primary independent variable. We used logistic regressions to examine retention, and linear regressions to analyze GPA. We also controlled for a range of student characteristics, including first-generation status, Pell Grant eligibility, and enrollment status (full-time or part-time), as well as demographic background factors such as gender and ethnicity. These controls help to account for differences in baseline characteristics that may influence academic outcomes, thus allowing us to isolate the impact of the CURE on GPA and retention. This approach improves the validity of our findings by reducing confounding effects, providing a clearer picture of how these courses contribute to student success.

### 4.6 Limitations

This study offers important insights into the impact of CUREs on student success and retention and into whether supporting faculty redesign of gatekeeping courses is an effective intervention to improve student success in STEM, but there are limitations in this study. First, the data were from a single research-intensive university, which means the findings may not fully apply to other types of institutions or contexts. Further studies should replicate this work at other institutions. Second, we focused on institutional indicators such as retention, persistence, and GPA, but did not examine the outcomes of faculty professional development, challenges faculty faced or how they navigated the implementation of CUREs in their courses. Future research should draw on multi-institutional samples to examine the challenges faculty encounter when implementing CUREs; logistics, financial, student resistance etc.; and the strategies they use to address them (Shortlidge and Brownell, 2016). As the field continues to grow, there is a need to evaluate the outcomes of CUREs for both students and faculty (Brownell and Kloser, 2015). This would provide deeper insight into how the fundamental tenets of CUREs in curriculum re-design can be effectively implemented to support student success in gatekeeping courses and improve retention across STEM pathways. This study also relies on institutional data so other factors such as intrinsic motivation, educational aspirations, and other socioemotional factors could not be included in the PSM model. The potential for selection bias is reduced by the fact that these courses were not advertised as CUREs, so it is unlikely that more motivated students enrolled in the CUREs, but it is still possible. Future research would benefit from using data that merges institutional data with survey data to be able to control for other student factors.

## 5 Findings

To answer the question of whether participation in a CURE improves student success when controlling for student background characteristics and early academic performance and when compared to like-peers, we ran a series of regressions on first- and second-year retention in college, first- and second-year retention in STEM fields, and cumulative GPA. Each regression was conducted on the full sample (all students in entering cohort 2022) and the matched sample of like-peers.

### 5.1 STEM retention

STEM first-year retention rates for students who participated in CUREs were 24% higher and 36.8% higher for second-year retention when compared to students who had not taken a CURE (Figure 2). CURE participants demonstrated substantially higher STEM first- and second-year retention. Specifically, CURE participants had a 91.4% first-year retention rate in STEM compared to 67.5% of students who did not take a CURE course. For second-year retention, STEM retention remained high among CURE participants (90.3%), whereas retention in the comparison group declined to 53.5%. In the matched sample, the comparison group had higher rates of transfer out of STEM as seen by the decrease in STEM retention rates and slight increase in overall retention rates (Figure 2).

**Figure 2:**
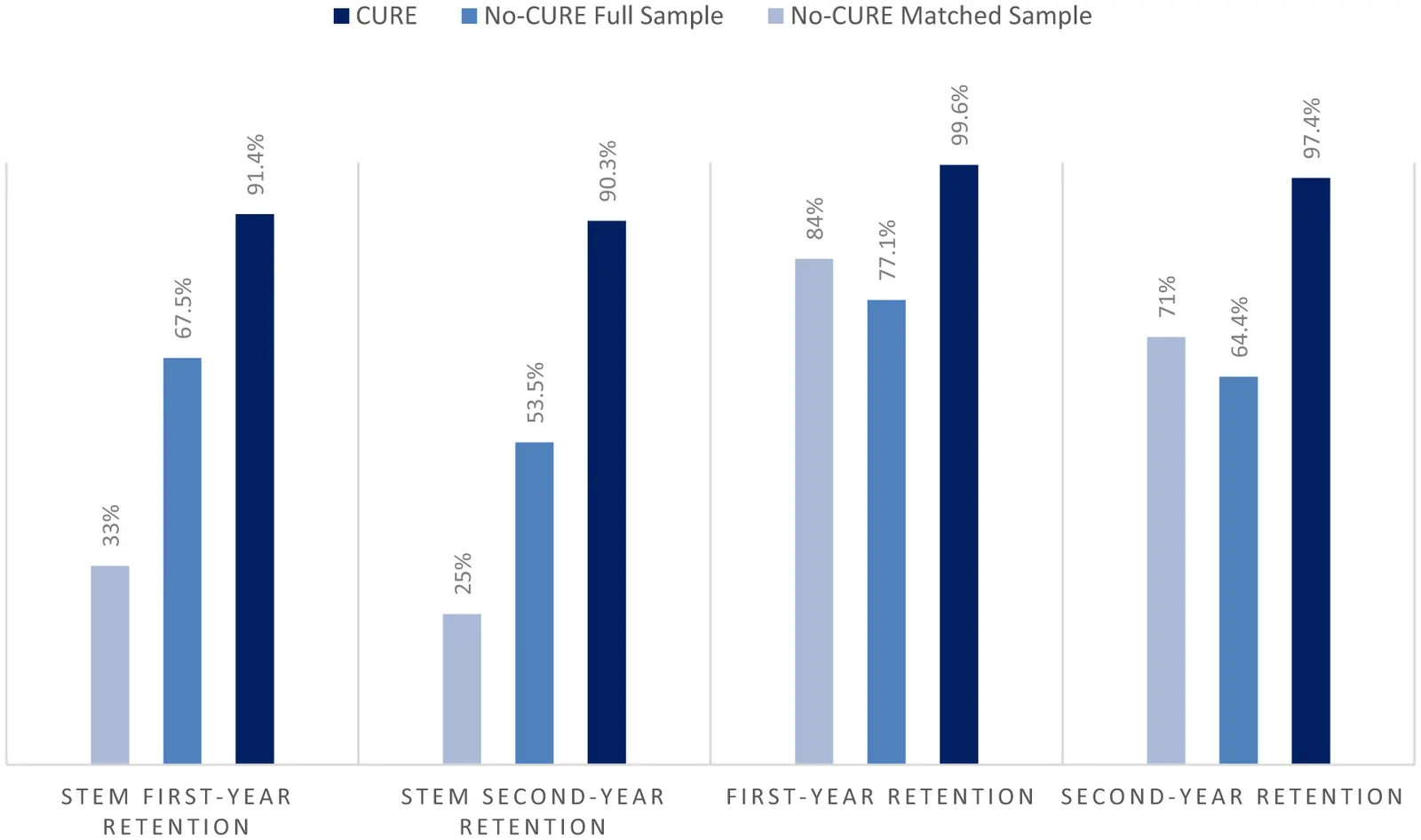
STEM Retention Rates and College Retention Rates for CURE Participants and Comparison Groups (Full Sample and Matched Sample).

To test these differences, logistic regressions were used for 1^st^-year and 2^nd^-year STEM retention (Table 2). CURE participation demonstrates a significant positive effect on first-year STEM retention (full sample, β = 0.194, *p* < 0.001). This finding indicates that participation in a CURE is associated with higher first-year retention rates in STEM. CURE's effect on second-year STEM retention is also positive and significant (full sample β = 0.346, *p* < 0.001), suggesting that the program supports students' academic engagement and persistence in STEM through the second year and into the third.

**Table 2:** Logistic regressions on CURE Participation and STEM retention.

Measure	Full sample	Full sample	Matched	Matched
	**1**^st^ **yr STEM ret**.	**2**^nd^ **yr STEM ret**.	**1**^st^ **yr STEM ret**.	**2**^ndyr^ **STEM ret**.
CURE	0.194^**^ (0.034)	0.346^**^ (0.036)	0.062^*^ (0.028)	0.201^**^ (0.034)
Fulltime	0.217^*^ (0.016)	0.246^*^ (0.017)	0.076^**^ (0.022)	0.143^**^ (0.027)
First-generation	−0.090^*^ (0.016)	−0.074^*^ (0.017)	−0.011 (0.020)	0.006 (0.025)
Pell	−0.013 (0.016)	−0.049^*^ (0.017)	0.020 (0.021)	−0.008 (0.026)
Gender male	0.030^*^ (0.014)	0.035^*^ (0.014)	0.044^**^ (0.017)	0.065^**^ (0.020)
Native American	−0.113 (0.058)	−0.048 (0.061)	0.079 (0.098)	0.057 (0.119)
Asian	0.067^*^ (0.029)	0.064^*^ (0.030)	0.050 (0.031)	0.047 (0.038)
Black	−0.096^*^ (0.031)	−0.132^*^ (0.033)	−0.021 (0.044)	−0.140^**^ (0.053)
Latine	0.014^*^ (0.017)	−0.020^*^ (0.018)	−0.009 (0.020)	−0.048 (0.024)
International	0.136^*^ (0.038)	0.091^*^ (0.040)	0.059 (0.046)	0.054 (0.056)
Pacific Islander	−0.201 (0.150)	−0.070 (0.158)	0.166^*^ (0.209)	0.289 (0.253)
Two or more races	−0.069^*^ (0.032)	−0.002^*^ (0.034)	−0.003 (0.039)	0.035 (0.047)
AR (2)	0.081	0.102	0.011	0.044
Prob > F	0.000	0.000	0.000	0.000
Obs	4,479	4,479	2,070	2,070

Standard errors are in parenthesis. ^*^p < 0.05; ^**^p < 0.001.

We also ran the same analyses on the matched sample of like-peers created through PSM. This allows us to further isolate the impact of CURE courses by comparing CURE students to students with similar first-semester GPAs and similar background characteristics (Table 2).

In the matched sample, CURE participation demonstrates a significant positive effect on first-year STEM retention (matched, β = 0.062, *p* < 0.05) though only marginally significant. Also on the matched sample, CURE's effect on second-year STEM retention is positive and significant (matched, β = 0.201, *p* < 0.001). This result highlights CURE's role in reinforcing academic engagement and persistence beyond the first year.

### 5.2 College retention

When looking at retention in college overall, we see a similar pattern of increased first-year and second-year retention rates (Figure 2). Logistic regression results also demonstrate a positive and significant effect on first-year retention (full sample, β = 0.166, *p* < 0.001) for CURE participants (Table 3). CURE participation is also significantly and positively related to second-year retention (full sample, β = 0.303, *p* < 0.001).

**Table 3:** Logistic Regressions on CURE Participation and College Retention.

**Measure**	Full sample	Full sample	Matched	Matched
	**1**^st^ **yr Retention**	**2**^nd^ **yr Retention**	**1**^st^ **yr Retention**	**2**^nd^ **yr Retention**
CURE	0.166^**^ (0.027)	0.303^**^ (0.031)	0.028 (0.015)	0.132^**^ (0.023)
Fulltime	0.179^**^ (0.009)	0.186^**^ (0.010)	0.025^**^ (0.009)	0.040^**^ (0.013)
First-generation	−0.107^**^ (0.009)	−0.133^**^(0.010)	−0.033^**^ (0.009)	−0.025 (0.014)
Pell	−0.005 (0.009)	−0.054^**^ (0.011)	0.028 (0.010)	−0.025 (0.014)
Gender male	−0.026^**^ (0.008)	−0.034^**^ (0.009)	0.002 (0.008)	−0.001 (0.011)
Native American	−0.125^**^ (0.034)	−0.144^**^ (0.038)	0.060 (0.041)	−0.018 (0.061)
Asian	0.044^*^ (0.018)	0.030 (0.021)	0.022 (0.016)	0.034 (0.023)
Black	−0.162^**^(0.018)	−0.197^**^ (0.020)	0.033 (0.022)	−0.063 (0.033)
Latine	0.003 (0.010)	−0.020 (0.011)	0.001 (0.009)	−0.030^*^ (0.013)
International	0.060^*^ (0.024)	−0.022 (0.028)	0.012 (0.023)	−0.062 (0.034)
Pacific Islander	−0.003 (0.081)	−0.079 (0.091)	0.058 (0.113)	−0.130 (0.168)
Two or more races	−0.031 (0.018)	−0.005 (0.020)	0.033 (0.018)	0.021 (0.026)
AR (2)	0.081	0.094	0.007	0.018
Prob > F	0.000	0.000	0.000	0.000
Obs	11,124	11,124	4,138	4,138

Standard errors are in parenthesis. ^*^p < 0.05; ^**^p < 0.001.

In the same analysis on the matched sample, CURE participation demonstrates a positive though non-significant effect on overall first-year retention (matched, β = 0.028, *p* < 0.1; Table 3) but positive and significant effect on second-year retention (matched, β = 0.132, *p* < 0.001).

### 5.3 Cumulative GPA

In addition to retention, we also explored the impact of CURE participation on cumulative GPA at the end of their second year of college (Figure 3). Students who completed a CURE in their first year had substantially higher GPA at the end of their second year; 3.38 average GPA for CURE participants, compared to 2.13 for students who did not take a CURE course in the full sample and 2.41 in the matched sample of like-peers.

**Figure 3:**
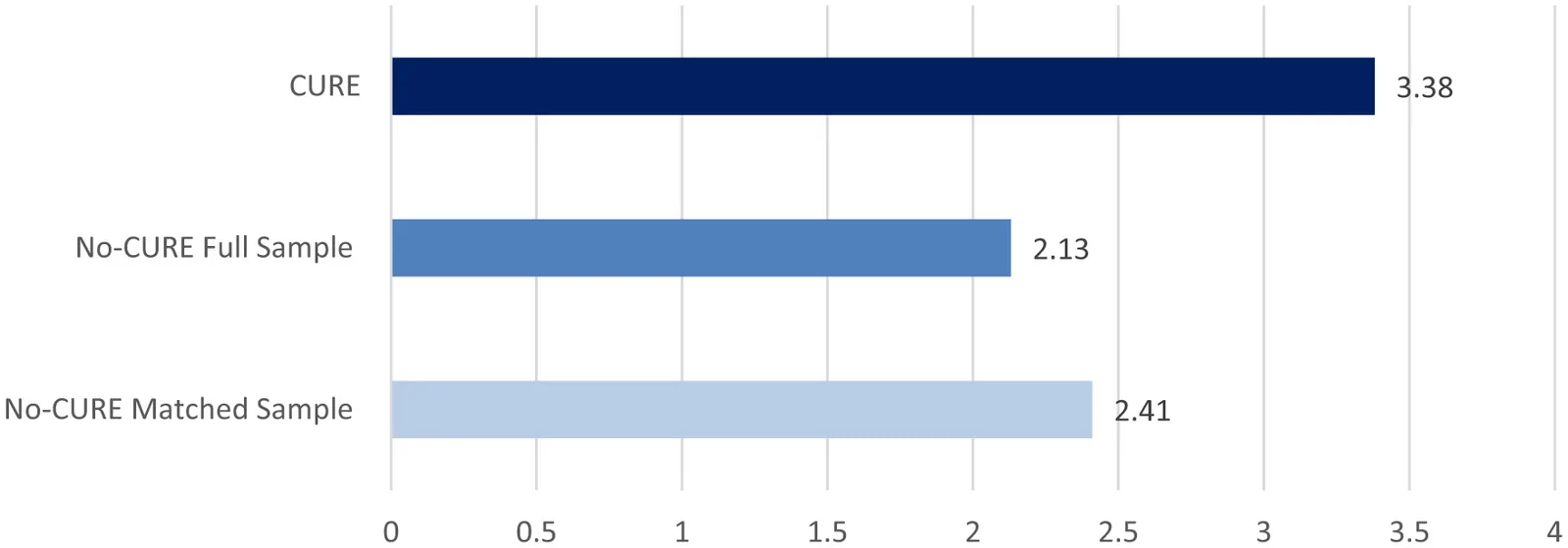
Cumulative GPA for CURE Participants and Non-participants (Full Sample and Matched Sample).

Linear regressions demonstrate significant positive effects on second-year CGPA (full sample, β = 1.054, *p* < 0.001; Table 4).

**Table 4:** Linear regressions on CURE participation and 2^nd^-year cumulative GPA.

**Variables**	Full sample	Matched
CURE	1.054^**^ (0.105)	0.462^**^ (0.085)
Fulltime	0.773^**^ (0.033)	0.410^**^ (0.047)
First-generation	−0.465^**^ (0.035)	−0.156^**^ (0.051)
Pell	−0.233^**^ (0.36)	−0.110^*^ (0.053)
STEM	0.006 (0.031)	−0.075 (0.040)
Gender	0.218^**^ (0.30)	−0.111^**^ (0.042)
Native American	−0.668^**^ (0.32)	−0.381 (0.225)
Asian	0.107^*^ (0.72)	0.098 (0.087)
Black	−0.793^**^ (0.70)	−0.500^**^ (0.121)
Latine	0.150^**^ (0.370)	−0.231 (0.500)
International	0.203^*^ (0.095)	−0.291^*^ (0.127)
Pacific Islander	−0.233 (0.314)	−0.641 (0.210)
Two or more races	−0.026 (0.069)	0.064 (0.098)
AR (2)	0.123	0.052
Prob > *F*	0.000	0.000
Obs	11,124	4,138

Standard errors are in parenthesis. ^*^p < 0.05; ^**^ *p* < 0.001.

These findings hold true even when comparing a matched sample of like-peers (Table 4). CURE participation is still significantly related to higher CGPA. CUREs have a positive and significant effect on second-year CGPA (matched, β = 0.462, *p* < 0.001). This finding indicates that participation in CURE enhances academic performance in addition to retention.

## 6 Conclusion

The study examined the impact of supporting faculty in redesigning courses as CUREs that foster inclusive, research-centered learning environments on student success and student retention in STEM programs. Findings show a consistent positive association between CURE participation and STEM persistence and success. It was found that students who participate in CUREs are more likely to be retained in STEM programs beyond the first year, and the association strengthens after the second year. In the matched analyses, CURE participation remains significantly related to higher second-year STEM retention and higher second-year cumulative GPA, indicating benefits that persist even when comparing students with similar observed likelihoods of enrolling in a CURE and similar early academic performance. These patterns suggest that integrating authentic research within credit-bearing coursework can support early persistence in STEM pathways, which has a significant impact on supporting student success and retention. By intentionally restructuring how students engage in gateway courses, institutions can redistribute access to high-impact research experiences and alter the conditions under which students succeed.

## 7 Discussion

This study examined how institutions can reshape introductory STEM courses through implementing CUREs to foster student success, and how this curricular shift can influence retention in STEM and higher education. The analysis focused on how implementing CUREs in gateway courses is associated with first- and second-year retention rates, first- and second-year retention rates in STEM fields, and cumulative GPAs. Using propensity score matching to create a *post-hoc*, quasi-experimental design, the results show a consistent pattern in which participation in CUREs is associated with significantly higher student success in STEM. In both the full and matched samples, students who participated in CUREs were more likely to remain in STEM after their first year, and this positive association became even stronger after the second year. These findings indicate that implementing CUREs in gateway courses supports persistence and academic performance, particularly in STEM disciplines as well as in other fields. These results align with Auchincloss et al. (2014) who argue that when students engage in authentic research through their coursework, they are more likely to persist in science and develop skills that matter for their long-term academic success and career trajectories. Auchincloss et al. (2014) frame CUREs as a scalable way to involve many more students in meaningful research and highlight persistence, scientific self-efficacy, and clarification of career goals as central outcomes. The positive relationship between CURE participation and STEM retention in this study also reaffirms work on large CURE development programs such as the CURE Training Institute at the University of Arizona. Using a similar propensity score matching method, Rodenbusch et al. (2016) found that students in a multi-semester CURE program under the Freshman Research Initiative were more likely than comparable peers to graduate with a STEM degree and to complete their degrees within 6 years, even when final GPAs did not differ at graduation. The present findings extend this literature by showing that CURE participation is tied not only to long-term degree milestones, but also to intermediate outcomes such as year-to-year STEM retention and second-year GPA. These results suggest that CUREs may shape students' trajectories at multiple points: in the short term, by helping students stay in their majors and remain enrolled at the institution, and over the longer period of the degree, by supporting continued academic success through faculty re-design of curriculum through CUREs. The findings also show that CUREs positively and significantly impact overall retention, underscoring the importance of integrating CUREs across the disciplines. Prior work has shown that undergraduate research experiences enhance students' academic performance, research abilities, and retention in STEM fields (Linn et al., 2015), reinforcing the importance of involving students in research projects to encourage them to pursue STEM-related education and employment. Bangera and Brownell (2014) argue that faculty redesigning gatekeeping courses through CUREs can make research experiences more inclusive by lowering barriers related to faculty selection, prior achievement, and social capital. They emphasize CUREs to reach students who are often excluded from traditional research apprenticeships, such as first-generation and racially minoritized students. Mentored (out-of-class) undergraduate research experiences have also been shown to have a compensatory effect where they are even more beneficial for minoritized students (Kinzie et al., 2008; Haeger et al., 2024). Future research should also explore whether CUREs may have a compensatory effect. Hernandez et al. (2018) used a large, multi-institutional, propensity score-matched design and found that sustained undergraduate research experiences broaden participation in the scientific workforce, particularly for underrepresented students, especially when the experiences are intensive and long term. Their work underscores that research experiences can be powerful, especially for marginalized students, yet they still operate within systems shaped by racism, class stratification, and uneven institutional support. The present findings resonate with that message. CUREs are clearly associated with better persistence and performance, but they do not, on their own, erase structural inequities in STEM and higher education. Hernandez et al. (2018) also illustrates when and how students engage in research matters. In their study, only students who spent 10 h a week over two semesters had significant benefits from their research experience. Additionally, recent research has questioned the efficacy of requiring all students to participate in a CURE. Laporte and Casper (2026) found a significant benefit to students who opted into taking a CURE in biology, but not to those who were required to take the course as a CURE. This study relied on data from a single course at a single instruction and compared voluntary CURE participation pre-pandemic to compulsory CURE enrollment during the pandemic (2021-2022). Despite these limitations, this study highlights the need to critically examine when and how CUREs are implemented throughout the curriculum.

In addition to the present study, a program evaluation of the CURE Training Institute also explored faculty experiences in developing and teaching CUREs (Kahlid et al., 2025). Faculty who participated in CURE training and re-designed their courses, including introductory gatekeeping courses, reported a clear shift in how they approach teaching. Rather than relying on traditional research experiences that are often less inclusive, faculty adopted more inclusive and engaging teaching practices that support broader student participation such as scaffolded research experiences, collaborative learning, and iterative feedback that emphasized process over performance (Kahlid et al., 2025). These changes reflect a growing recognition that persistence in STEM is not only a function of individual ability but is also shaped by curricular design and classroom climate. These equity-focused approaches were designed to ensure that all students, including minoritized populations, have meaningful opportunities to succeed in STEM courses and persist along STEM pathways. This shift also challenged deficit-oriented assumptions about student preparedness for STEM. Gatekeeping courses have historically been used to separate students based on perceived grit and ability. In contrast, faculty began to reframe all students as capable of engaging in authentic research when provided with the appropriate structure and support. According to Hatfield et al. (2022), low performance in introductory STEM courses was more strongly associated with failure to earn a STEM degree among underrepresented minority (URM) students than among their peers, even after accounting for academic preparation and initial STEM intentions. These findings reveal that gateway courses may contribute to inequities in STEM persistence and highlight the need to explore curricular changes to improve gateway courses.

At the institutional level, the implementation of CUREs at the University of Arizona demonstrated that high-impact, equity-oriented practices can effectively support student success and retention, particularly early in students' academic pathways. In summary, the findings provide strong grounds to argue that CUREs offer an impactful, systemic intervention for student success and retention rather than merely a pedagogical strategy for undergraduate education. Students who participate in CUREs are more likely to persist in STEM majors and to remain enrolled in their programs while maintaining stronger academic performance, such as higher GPAs. These outcomes are foundational for student success pathways, such as staying in a program, completing a degree, and earning a strong GPA to support student success and retention. Institutional efforts demonstrate that this form of organizational redesign is achievable at scale. When institutions couple deliberate course redesign with robust faculty support and evidence-based refinement, they fundamentally alter the landscape of opportunity, enabling thousands of additional students to succeed in foundational courses and progress toward degree completion. This represents not merely improved pedagogy, but institutional transformation to reshape how access, competence, and academic standards intersect.

## 8 Implications

This study offers substantial evidence that institutional redesign of gatekeeping mechanisms through the transformation of foundational courses from gatekeepers to gateways represents a powerful lever for expanding educational opportunity without compromising academic rigor. Rather than treating CUREs as simply an additive pedagogical intervention, this suggests that implementing CUREs in gateway courses can be an effective educational intervention for improving undergraduate education. Institutions also must consider when and how CUREs are implemented. To capitalize on this potential for systemic change, institutions must attend to three critical dimensions of implementation. First, institutions should offer CUREs early in students‘ academic programs to provide experiential learning opportunities that will support students' persistence, retention, and success. These first- and second-year CUREs should be intentionally designed to engage students from marginalized backgrounds, ensuring they gain early exposure to research that promotes student success in gateway courses and prepares them for success on STEM pathways. Second, it is important to examine which components of CURE courses are most critical for student success and retention to design more efficient educational interventions to support students in STEM programs. Prior research has suggested that requiring all students in a major to take a CURE (compulsory engagement) is less effective than letting students choose between more traditional lab courses and CUREs (Laporte and Casper, 2026). This research was conducted at a single institution, and the compulsory CURE course was offered in 2021-2022 compared to the traditional course and the volunteer conditions which were all pre-pandemic. Future research should further test under what conditions CUREs are most effective and whether requiring students to take a CURE is as effective as voluntary engagement. Third, and fundamentally, institutions must commit sustained resources to faculty development as a form of organizational infrastructure. Institutions should provide adequate funding for professional development to equip faculty and staff with the skills needed to implement inclusive and equity-oriented pedagogical approaches such as CUREs. Institutions should invest in professional development initiatives, such as the CUREs Training Institute at the University of Arizona, to support teaching practices that promote student access, success, belonging, and retention in STEM programs. By supporting faculty in fostering inclusive and supportive environments, faculty teaching CUREs can help bridge gaps in undergraduate education and build stronger workforce development competencies among undergraduate students.

## Data Availability

The data analyzed in this study is subject to the following licenses/restrictions: we used institutional student records, so they are not publicly available. We received institutional approval to use a de-identified version of this data, but are not permitted to share the raw data. Requests to access these datasets should be directed to heather3@arizona.edu.
